# Supplementary material for: From toxicogenomics data to cumulative assessment groups: a framework for chemical grouping
Source: Arch Toxicol. 2025 Sep 26;100(1):173–91. doi: 10.1007/s00204-025-04133-w (PMC12858531; doi:10.1007/s00204-025-04133-w)
Supplement: Supplementary file 1 — (pdf 529 KB) [file 204_2025_4133_MOESM1_ESM.pdf]

Supplementary Material

From Toxicogenomics Data to Cumulative Assessment Groups: A Framework for Chemical Grouping

Sebastian Canzler · Julienne Lehmann · Jana Schor · Wibke Busch · Giovanni Iacono · Jörg Hackermüller

S.Canzler · J.Lehmann · J.Schor · J.Hackermüller
Department of Computational Biology and Chemistry, Helmholtz Centre for Environmental Research - UFZ, 04318 Leipzig, Germany
E-mail: sebastian.canzler@ufz.de

W. Busch
Department Ecotoxicology Helmholtz Centre for Environmental Research - UFZ, 04318 Leipzig, Germany

J. Lehmann
HIV Cure Research Center, Department of Internal Medicine and Pediatrics, Ghent University Hospital, Ghent University, 9000 Ghent, Belgium

G. Iacono
European Food Safety Authority (EFSA), 43126 Parma, Italy

J. Hackermüller
Department of Computer Science, Leipzig University, 04109 Leipzig, Germany

S1 Data Sources

Table S1: Summary of source files that have been used to gather CTD, PubChem, and NCBI data.

Source	Property	File URL
CTD	Chem - Gene	https://ctdbase.org/reports/CTD_chem_gene_ixns.tsv.gz
CTD	Chem - Gene Types	https://ctdbase.org/reports/CTD_chem_gene_ixn_types.tsv
CTD	Chem - Disease	https://ctdbase.org/reports/CTD_chemicals_diseases.tsv.gz
CTD	Chem - Pathway	https://ctdbase.org/reports/CTD_chem_pathways_enriched.tsv.gz
CTD	Chem - GO	https://ctdbase.org/reports/CTD_chem_go_enriched.tsv.gz
CTD	Gene - Pathway	https://ctdbase.org/reports/CTD_genes_pathways.tsv.gz
CTD	Disease - Pathway	https://ctdbase.org/reports/CTD_diseases_pathways.tsv.gz
CTD	Chemicals	https://ctdbase.org/reports/CTD_chemicals.tsv.gz
CTD	Diseases	https://ctdbase.org/reports/CTD_diseases.tsv.gz
CTD	Genes	https://ctdbase.org/reports/CTD_genes.tsv.gz
CTD	Pathways	https://ctdbase.org/reports/CTD_pathways.tsv.gz
CTD	Anatomy terms	https://ctdbase.org/reports/CTD_anatomy.tsv.gz
CTD	Exposure	https://ctdbase.org/reports/CTD_exposure_events.tsv.gz
CTD	Exp. studies	https://ctdbase.org/reports/CTD_exposure_studies.tsv.gz
CTD	Chem - Phenotype	https://ctdbase.org/reports/CTD_pheno_term_ixns.tsv.gz
CTD	Gene - Disease	https://ctdbase.org/reports/CTD_genes_diseases.tsv.gz
PubChem	InChI key	https://ftp.ncbi.nlm.nih.gov/pubchem/Compound/Extras/CID-InChI-Key.gz
PubChem	SMILES	https://ftp.ncbi.nlm.nih.gov/pubchem/Compound/Extras/CID-SMILES.gz
PubChem	Molec. mass, Formula	https://ftp.ncbi.nlm.nih.gov/pubchem/Compound/Extras/CID-Mass.gz
PubChem	Title	https://ftp.ncbi.nlm.nih.gov/pubchem/Compound/Extras/CID-Title.gz
PubChem	CAS, ChEBI DTXSID	https://ftp.ncbi.nlm.nih.gov/pubchem/Compound/Extras/CID-Synonym-filtered.gz
NCBI	gene mapping	https://ftp.ncbi.nlm.nih.gov/gene/DATA/gene_orthologs.gz
NCBI	Gene - Penotype	https://ftp.ncbi.nlm.nih.gov/gene/DATA/gene2go.gz

Table S2: Table summarizing the SQLite structure. The data in the tables starting with 'CID' was collected from PubChem and originated from different source files. Tables starting with 'CTD' originated from CTD. Table names are equivalent to the CTD file name. The snapshot date of the data set was March 20th, 2023.

Table name	# Columns	# Entries
CID_chemicalproperties	6	12041
CID_IDs	6	16332
CTD_anatomy	9	1844
CTD_chem_gene_ixns	12	2487743
CTD_chem_gene_ixn_types	4	53
CTD_chem_go_enriched	13	6237303
CTD_chemicals	8	175847
CTD_chemicals_diseases	10	8276711
CTD_chem_pathways_enriched	11	1483203
CTD_diseases	9	13270
CTD_diseases_pathways	5	609539
CTD_exposure_events	43	204735
CTD_exposure_studies	10	3304
CTD_genes	8	589897
CTD_genes_diseases	9	100715108
CTD_genes_pathways	4	135783
CTD_pathways	2	2567
CTD_pheno_term_ixns	13	340861

Table S3: Summary of the Entrez gene ID mapping between the species *Homo sapiens*, *Mus musculus*, and *Rattus norvegicus*. The table shows the total number of genes that are listed in chemical-gene interactions and the number of *primary* gene IDs that originate from either human, mouse, or rat. The last column shows the number of primary gene IDs that could not be assigned to the organism of interest.

Organism	Genes	Primary IDs from				IDs without mapping
		H.sapiens	*M.musculus*	*R.norvegicus*	other	
H.sapiens	27354	26253	265	36	800	713
M.musculus	22673	17261	4808	52	552	600
R.norvegicus	21691	17638	1977	1612	464	1098

S2 Tissue groups

Adrenal gland

- Adrenal Cortex
- Adrenal Glands

Bone marrow

- Mesenchymal Stem Cells
- Myeloid Cells
- Bone Marrow
- Endothelial Progenitor Cells
- Cancellous Bone
- Bone Marrow Cells

Bones/skeleton

- Osteoblasts
- Skull
- Synovial Membrane
- Bone and Bones
- Jaw
- Maxilla
- Orbit
- Turbinates
- Nucleus Pulposus
- Lumbar Vertebrae
- Cartilage
- Articular
- Ankle Joint
- Joint Capsule
- Knee Joint
- Osteocytes
- Femur
- Leg Bones
- Tibia
- Spine
- Joints
- Cartilage, Articular
- Menisci, Tibial
- Skeleton
- Growth Plate
- Mandible
- Tarsal Joints
- Periosteum

Cardiovascular system

- Cardiovascular System
- Blood Vessels
- Exudates and Transudates
- Arteries
- Aorta
- Brachial Artery
- Middle Cerebral Artery
- Coronary Vessels
- Coronary Sinus
- Umbilical Arteries
- Heart
- Heart Atria
- Heart Ventricles
- Myocardium
- Myocytes, Cardiac
- Endothelial Cells
- Human Umbilical Vein Endothelial Cells
- Mesenteric Arteries
- Pulmonary Artery
- Microvessels
- Endothelium, Vascular
- Veins
- Umbilical Veins
- Aortic Valve
- Myoblasts, Cardiac
- Blood-Brain Barrier
- Aorta, Abdominal
- Aorta, Thoracic
- Sinus of Valsalva
- Brachiocephalic Trunk
- Carotid Arteries
- Femoral Artery
- Mesenteric Artery, Superior
- Uterine Artery
- Venules
- Cerebral Veins
- Hepatic Veins
- Portal Vein
- Ductus Arteriosus
- Pericardium
- Mitochondria, Heart
- Blood-Testis Barrier
- Adventitia
- Arterioles
- Basilar Artery
- Cerebral Arteries
- Anterior Cerebral Artery
- Circle of Willis
- Meningeal Arteries
- Thoracic Arteries
- Capillaries
- Retinal Vessels
- Tunica Intima
- Portal System
- Mesenteric Veins

Eye

- Eye
- Retinal Pigment Epithelium
- Conjunctiva
- Cornea
- Epithelium, Corneal
- Lens, Crystalline
- Trabecular Meshwork
- Retina
- Photoreceptor Cells
- Retinal Ganglion Cells
- Tenon Capsule
- Uvea
- Iris
- Lacrimal Apparatus
- Photoreceptor Cells, Vertebrate
- Retinal Neurons
- Retinal Horizontal Cells
- Ciliary Body
- Corneal Stroma

Gallbladder

- Gallbladder

Haematological system

- Granulocyte Precursor Cells
- Lymphoid Progenitor Cells
- Myeloid Progenitor Cells
- Granulocyte-Macrophage Progenitor Cells
- U937 Cells
- Erythroid Precursor Cells
- Blood
- Blood Cells
- Serum
- Blood Buffy Coat
- Plasma
- Erythrocytes
- Blood Platelets
- Fetal Blood
- Erythrocyte Membrane
- Hematopoietic Stem Cells
- Precursor Cells, B-Lymphoid
- Precursor Cells, T-Lymphoid
- Erythroblasts
- Megakaryocyte Progenitor Cells
- Megakaryocytes
- Reticulocytes
- Erythroid Cells
- Thymocytes

Kidney

- Madin Darby Canine Kidney Cells
- Kidney
- Kidney Glomerulus
- Mesangial Cells
- Kidney Medulla
- Kidney Tubules, Proximal
- Kidney Cortex
- Podocytes
- Kidney Tubules
- Juxtaglomerular Apparatus
- Kidney Tubules, Collecting
- Kidney Tubules, Distal

Liver

- Hep G2 Cells
- Microsomes, Liver
- Hepatic Stellate Cells
- Hepatocytes
- Bile Ducts
- Liver
- Bile Ducts, Intrahepatic
- Bile Canaliculi
- Mitochondria, Liver
- Bile

Muscles

- Muscle, Skeletal
- Muscle, Smooth, Vascular
- Muscles
- Muscle, Striated
- Satellite Cells, Skeletal Muscle
- Muscle Cells
- Myoblasts, Skeletal
- Myocytes, Smooth Muscle
- Rectus Abdominis
- Quadriceps Muscle
- Diaphragm
- Muscle Fibers, Skeletal
- Myoblasts
- Abdominal Muscles
- Tendons
- Achilles Tendon

Nervous system

- Nervous System
- Corticotrophs
- PC12 Cells
- Brain
- Substantia Nigra
- Ventral Tegmental Area
- Hippocampus
- CA1 Region, Hippocampal
- Parahippocampal Gyrus
- Entorhinal Cortex
- Corpus Striatum
- Nucleus Accumbens
- Cerebral Cortex
- Frontal Lobe
- Neocortex
- Spinal Cord
- Astrocytes
- Oligodendroglia
- Neurons
- Dopaminergic Neurons
- Neural Stem Cells
- Mesencephalon
- Cerebellum
- CA2 Region, Hippocampal
- CA3 Region, Hippocampal
- Dentate Gyrus
- Somatotrophs
- Cerebrum
- Meninges
- Neuroglia
- Ependymoglial Cells
- Microglia
- Schwann Cells
- Peripheral Nervous System
- Sympathetic Nervous System
- Neuroepithelial Cells
- Central Nervous System
- Brain Stem
- Cerebral Peduncle
- Pars Compacta
- Tegmentum Mesencephali
- Locus Coeruleus
- Inferior Colliculi
- Superior Colliculi
- Cerebellar Cortex
- Purkinje Cells
- Pons
- Facial Nucleus
- Superior Olivary Complex
- Medulla Oblongata
- Cerebral Ventricles
- Lateral Ventricles
- Limbic System
- Amygdala
- Pineal Gland
- Fornix, Brain
- Hypothalamus
- Paraventricular Hypothalamic Nucleus
- Arcuate Nucleus of Hypothalamus
- Median Eminence
- Gonadotrophs
- Thalamus
- Telencephalon
- Globus Pallidus
- Neostriatum
- Putamen
- Motor Cortex
- Prefrontal Cortex
- Somatosensory Cortex
- Basal Forebrain
- Olfactory Bulb
- Septal Nuclei
- Corpus Callosum
- White Matter
- Pyramidal Tracts
- Spinal Cord Dorsal Horn
- Spinal Cord Ventral Horn
- Ganglia, Spinal
- Olfactory Pathways
- Medial Forebrain Bundle
- Oligodendrocyte Precursor Cells
- Cholinergic Neurons
- Neurites
- GABAergic Neurons
- Hair Cells, Auditory
- Sensory Receptor Cells
- Motor Neurons
- Pyramidal Cells
- Synapses
- Anterior Horn Cells
- Hair Cells, Auditory, Inner
- Hair Cells, Auditory, Outer
- Olfactory Receptor Neurons
- Myenteric Plexus
- Optic Nerve
- Sciatic Nerve
- Neuroendocrine Cells
- Raphe Nuclei
- Dorsal Raphe Nucleus
- Trigeminal Nucleus, Spinal
- Olivary Nucleus
- Solitary Nucleus
- Choroid Plexus
- Basolateral Nuclear Complex
- Central Amygdaloid Nucleus
- Corticomedial Nuclear Complex
- Preoptic Area
- Supraoptic Nucleus
- Gyrus Cinguli
- Prosencephalon
- Geniculate Bodies
- Lateral Thalamic Nuclei
- Midline Thalamic Nuclei
- Basal Ganglia
- Parietal Lobe
- Sensorimotor Cortex
- Piriform Cortex
- Temporal Lobe
- Posterior Horn Cells
- Dura Mater
- Pia Mater
- Cervical Cord
- Spiral Ganglion
- Myelin Sheath
- Dendritic Spines
- Autonomic Pathways
- Peripheral Nerves
- Spinal Nerves
- Stellate Ganglion
- Superior Cervical Ganglion
- Carotid Body
- Vagus Nerve
- Spinal Nerve Roots

Parathyroid gland

- Parathyroid Glands

Reproductive system

- Semen
- Placenta
- Endometrium
- Trophoblasts
- Spermatozoa
- Ovary
- Granulosa Cells
- Testis
- Prostate
- PC-3 Cells
- Genitalia, Male
- Penis
- Genitalia, Female
- Oocytes
- Follicular Fluid
- Luteal Cells
- Cervix Uteri
- Ovarian Follicle
- Foreskin
- Mesoderm
- Uterus
- Germ Cells
- Sertoli Cells
- Chorionic Villi
- Cumulus Cells
- Decidua
- Leydig Cells
- Spermatocytes
- Epididymis
- Seminiferous Tubules
- Spermatogonia
- Seminal Vesicles
- Theca Cells
- Gonads
- Zygote
- Vagina
- Spermatids
- Corpus Luteum
- Seminiferous Epithelium
- Sperm Head
- Rete Testis
- Vas Deferens
- Fallopian Tubes
- Urethra
- Genitalia

Breast

- Mammary Glands
- Mammary Glands, Human
- Mammary Glands, Animal
- MCF-7 Cells
- Breast

Spleen

- Spleen

Thyroid gland

- Thyroid Epithelial Cells
- Thyroid Gland

Urinary bladder

- Urothelium
- Urinary Bladder
- Urinary Tract
- Ureter
- Urothelium

Gastrointestinal tract

- Gastrointestinal Tract
- Colon
- Intestine, Small
- Duodenum
- Mouth
- Taste Buds
- Stomach
- Dental Pulp
- Caco-2 Cells
- HCT116 Cells
- Intestines
- Intestine, Large
- Cecum
- Rectum
- Submandibular Gland
- Tongue
- Pharynx
- Esophagus
- Esophageal Mucosa
- Gastric Mucosa
- Periodontium
- Gingiva
- Periodontal Ligament
- Tooth
- Bicuspid
- Molar
- Dental Papilla
- Hypopharynx
- Nasopharynx
- Peritoneal Cavity
- Mouth Floor
- Omentum
- Intestinal Mucosa
- Ileum
- Jejunum
- Lower Gastrointestinal Tract
- Upper Gastrointestinal Tract
- Dental Cementum
- Enamel Organ
- Enteroendocrine Cells
- Enterocytes
- Incisor
- Dental Enamel
- Parietal Cells, Gastric
- HT29 Cells
- Interstitial Cells of Cajal

Immune system

- RAW 264.7 Cells
- Osteoclasts
- Synoviocytes
- Immune System
- B-Lymphocytes
- B-Lymphocyte Subsets
- Plasma Cells
- Dendritic Cells
- Langerhans Cells
- Leukocytes
- Granulocytes
- HL-60 Cells
- Basophils
- Eosinophils
- Neutrophils
- Leukocytes, Mononuclear
- Lymphocytes
- Monocytes
- Endothelium, Lymphatic
- Mast Cells
- Macrophages
- Foam Cells
- Killer Cells, Natural
- T-Lymphocytes, Helper-Inducer
- T-Lymphocytes, Regulatory
- T-Lymphocytes
- CD4-Positive T-Lymphocytes
- CD8-Positive T-Lymphocytes
- Jurkat Cells
- Th17 Cells
- Lymphatic System
- Lymphoid Tissue
- Histiocytes
- Th1 Cells
- Th2 Cells
- Kupffer Cells
- Macrophages, Alveolar
- Macrophages, Peritoneal
- Phagocytes
- Myeloid-Derived Suppressor Cells
- Lymph
- THP-1 Cells

Lung

- A549 Cells
- Sputum
- Lung
- Bronchi
- Alveolar Epithelial Cells
- Bronchioles
- Pulmonary Alveoli
- Pleura
- Trachea
- Respiratory System

Lymph Nodes

- Lymph Nodes
- Germinal Center

Pancreas

- Islets of Langerhans
- Insulin-Secreting Cells
- Pancreatic Ducts
- Glucagon-Secreting Cells
- Pancreas
- Pancreas, Exocrine
- Pancreatic Stellate Cells

Pituitary gland

- Pituitary Gland
- Pituitary Gland, Anterior

Salivary gland

- Saliva
- Salivary Glands
- Parotid Gland

Skin

- HaCaT Cells
- Keratinocytes
- Melanocytes
- Skin
- Dermis
- Epidermis
- Hair Follicle
- Sebaceous Glands
- Epidermal Cells

Thymus

- Thymus Gland

Fat tissues

- Adipocytes
- Adipocytes, White
- Adipocytes, Brown
- Adipose Tissue
- Adipose Tissue, White
- Adipose Tissue, Brown
- Subcutaneous Fat
- Subcutaneous Tissue
- Intra-Abdominal Fat
- 3T3-L1 Cells

Embrionic stem cells

- HEK293 Cells
- Embryonic Stem Cells, Cultured
- Human Embryonic Stem Cells
- Embryonic Structures
- Mouse Embryonic Stem Cells
- Embryonic Germ Cells
- Embryonal Carcinoma Stem Cells

Table S4: Pesticide-related MeSH terms that were used to identify pesticides in `CTD`.

MeSH Term	Number of pesticides in `CTD`
Insecticides	151
Herbicides	78
Fungicides	63
Pesticides	19
Rodenticides	10
Insect Repellents	9
Chemosterilants	5
Molluscacides	3
Defoliants	3
Acaricides	2
Pesticide Synergists	1

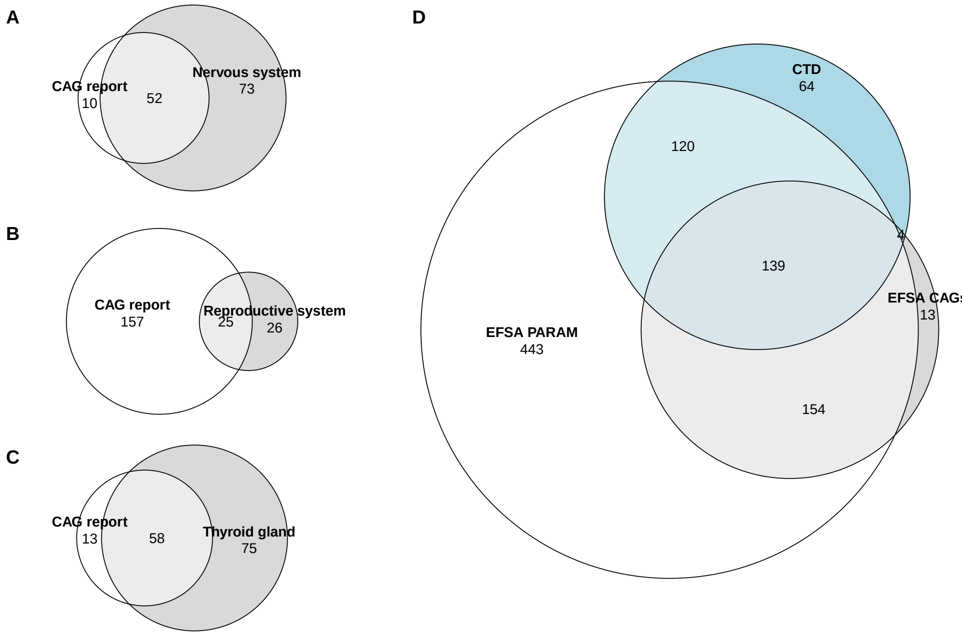

Fig. S1: Overlap of pesticides collected from different sources. Subfigures A-C focus on tissue-specific overlaps between CAGs initially proposed by Nielsen et al. (2012) and those subsequently established by EFSA [2019,2019a,2022]. Pesticides collected from the original CAG report in 2012 are denoted as 'CAG report' in those subfigures. (A) Pesticides impacting the nervous system. (B) Pesticides impacting the reproductive system and the updated report focusing on the craniofacial alterations. (C) Pesticides impacting the thyroid gland. (D) Overlap of pesticides collected from the EFSA PARAM catalogue, the combined CAG reports, and pesticides collected from `CTDbase` using the MeSH dictionary.

Table S5: MeSH terms that were listed in the MeSH Browser tree view as children of "Physiological Effects of Drugs [D27.505.696]", which are associated with pharmaceuticals.

MeSH Term	MeSH Term
Physiological Effects of Drugs	Abuse-Deterrent Formulations
Aversive Agents	Antipyretics
Antispermatogenic Agents	Sperm Immobilizing Agents
Spermatocidal Agents	Spermatogenesis-Blocking Agents
Astringents	Bone Density Conservation Agents
Calcium-Regulating Hormones and Agents	Calcium Channel Agonists
Calcium Channel Blockers	Central Nervous System Depressants
Anesthetics	Anesthetics, Combined
Anesthetics, General	Anesthetics, Inhalation
Anesthetics, Intravenous	Anesthetics, Dissociative
Anesthetics, Local	Hypnotics and Sedatives
Sleep Aids, Pharmaceutical	Orexin Receptor Antagonists
Narcotics	Analgesics, Opioid
Tranquilizing Agents	Anti-Anxiety Agents
Antimanic Agents	Antipsychotic Agents
Central Nervous System Stimulants	Aphrodisiacs
Appetite Stimulants	Convulsants
Cerumenolytic Agents	Emetics
Endocrine Disruptors	Galactogogues
Growth Substances	Angiogenesis Modulating Agents
Angiogenesis Inducing Agents	Angiogenesis Inhibitors
Growth Inhibitors	Angiogenesis Inhibitors
Plant Growth Regulators	Hallucinogens
Hormones, Hormone Substitutes, and Hormone Antagonists	Hormone Antagonists
Androgen Antagonists	Androgen Receptor Antagonists
Nonsteroidal Anti-Androgens	Antithyroid Agents
Calcimimetic Agents	Estrogen Antagonists
Aromatase Inhibitors	Estrogen Receptor Antagonists
Estrogen Receptor Modulators	Selective Estrogen Receptor Modulators
Insulin Antagonists	Leukotriene Antagonists
Mineralocorticoid Receptor Antagonists	Prostaglandin Antagonists
Puberty Inhibitors	Steroid Synthesis Inhibitors
14-alpha Demethylase Inhibitors	5-alpha Reductase Inhibitors
Aromatase Inhibitors	Hormones
Anabolic Agents	Androgens
Cannabinoid Receptor Modulators	Cannabinoid Receptor Agonists
Cannabinoid Receptor Antagonists	Estrogens
Estrogens, Non-Steroidal	Phytoestrogens
Glucocorticoids	Incretins
Mineralocorticoids	Progestins

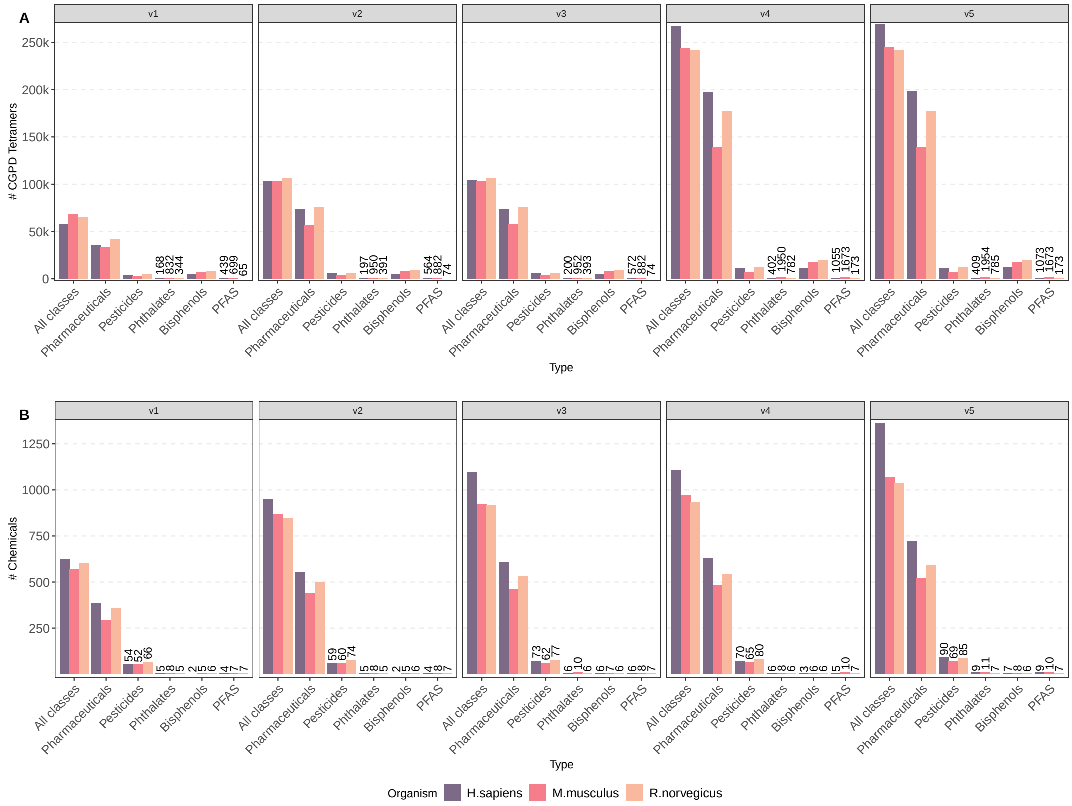

Fig. S2: **Subsetting `CTD`-derived data to different chemical classes.** (A) Number of CGPD tetramers calculated from `CTDbase`. (B) Number of chemicals for which CGPD tetramers could be calculated. tetramers have been individually calculated for the three target organisms human, rat, and mouse, and five individual filtering strategies - facets 'v1' to 'v5'.. Pesticides have been collected from the EFSA PARAM catalogue (denoted as EFSA Pesticides), from four different CAG reports (EFSA CAGs) and the `CTDbase` itself based on the MeSH dictionary (CTD Pesticides). Pharmaceuticals have been collected from the Drugbank and the `CTDbase` itself based on the MeSH dictionary. Bisphenols, Phthalates, and PFAS have been collected from `CTDbase` using the MeSH dictionary.

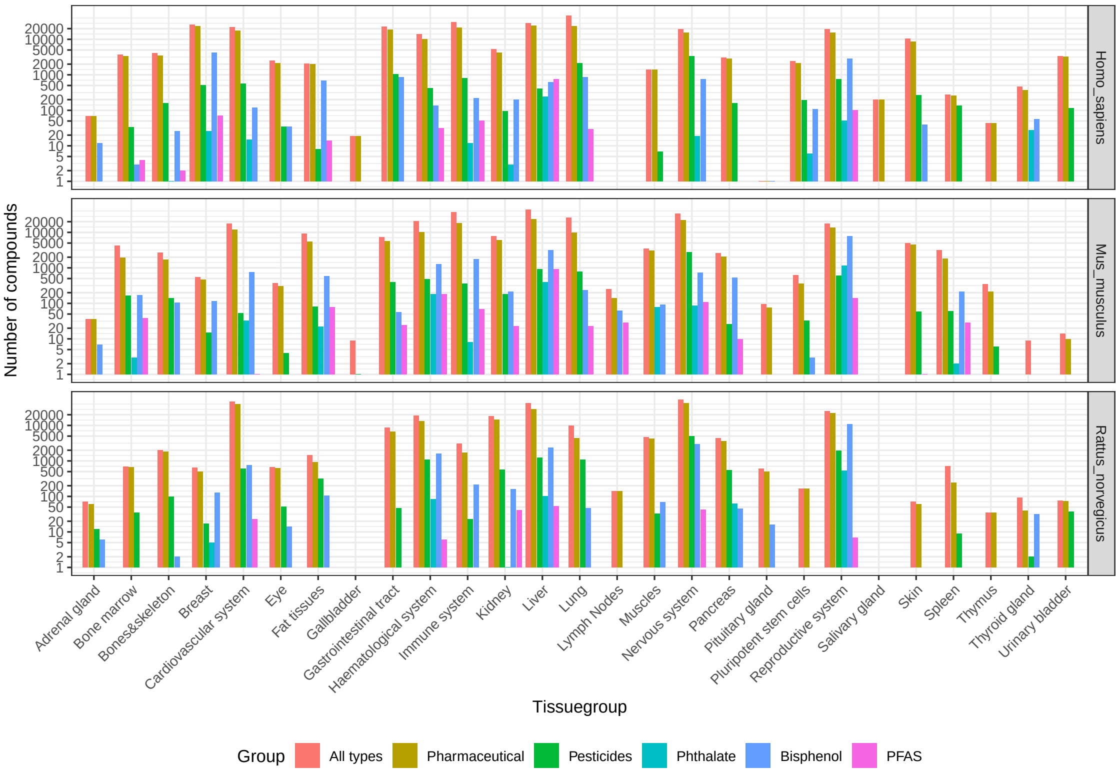

Fig. S3: **Visualizing CGPD tetramers per tissue** The number of CGPD tetramers that were calculated for a specific tissuegroup is shown for filtering strategy v5. Tetramers have be classified in distinct chemical classes. The analysis was visualized separately for the three target organisms.

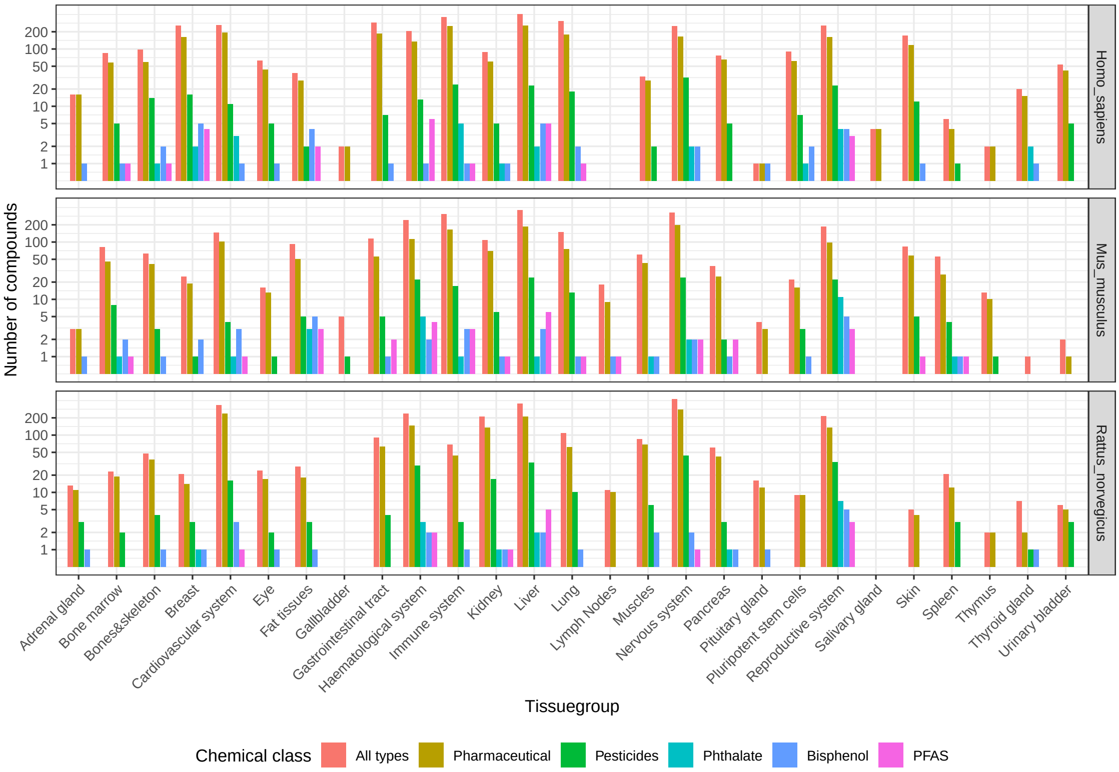

Fig. S4: **Visualizing the number of chemicals per tissue** The number of compounds that effect a specific tissuegroup is shown for filtering strategy v5. Chemicals have be classified in distinct chemical classes. The analysis was visualized separately for the three target organisms.

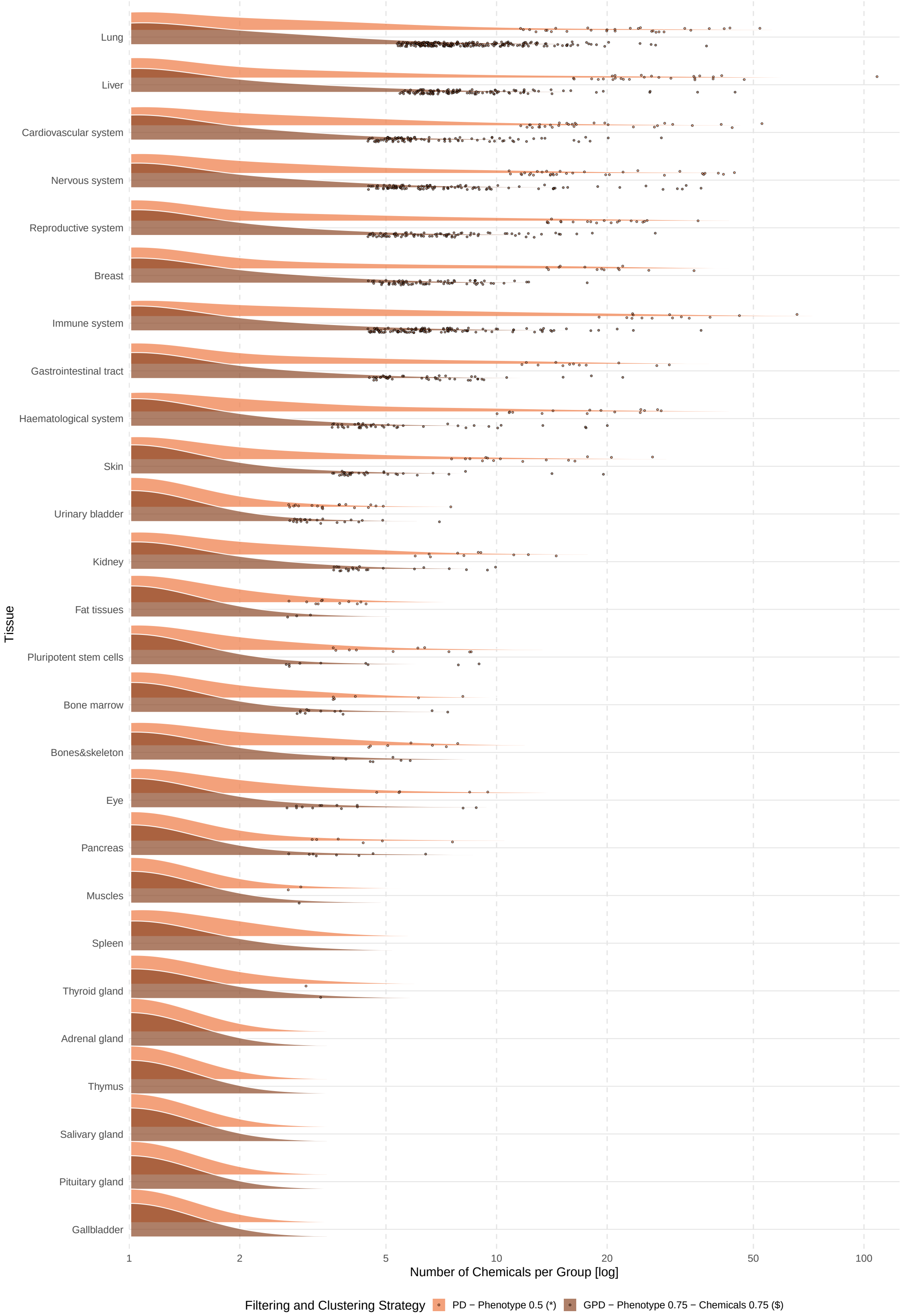

Fig. S5: **Comparison of the number of chemicals per group.** Ridge plots indicate the density of the number of chemicals per chemical group for each tissue group based on two different filtering and clustering strategies. First, a very stringent method based on PD grouping and phenotype-based clustering with a threshold of 0.5 (marked with $*$ in Figure 4 A). Second, a grouping strategy utilizing GPD grouping and a clustering based on phenotypes and chemicals with more relaxed thresholds of 0.75 (marked with \$ in Figure 4 A) . Individual points represent groups exceeding the 95th percentile within their respective distribution (right-tail outliers).

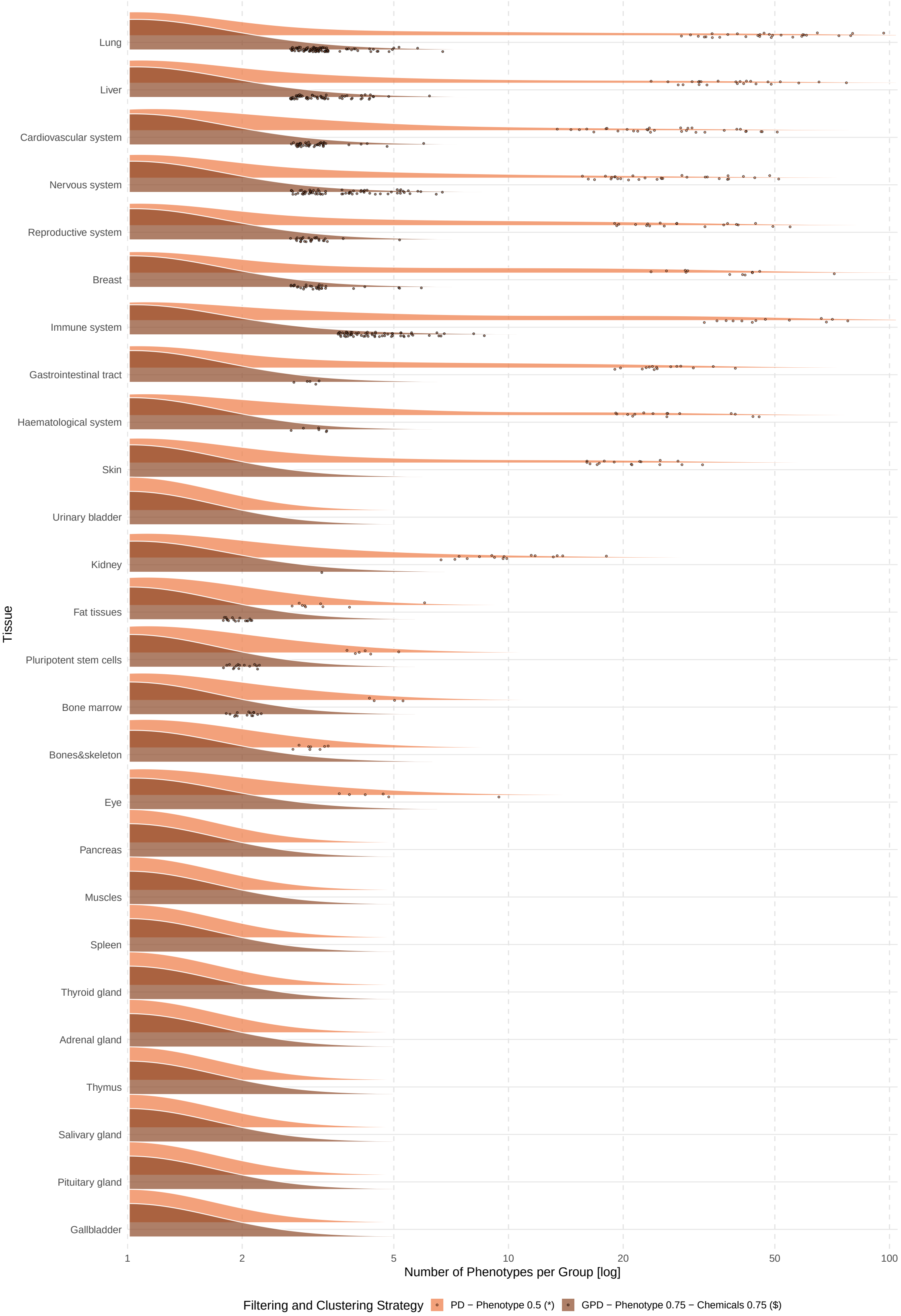

Fig. S6: **Comparison of the number of phenotypes per group.** Ridge plots indicate the density of the number of phenotypes per chemical group for each tissue group based on two different filtering and clustering strategies. First, a very stringent method based on PD grouping and phenotype-based clustering with a threshold of 0.5 (marked with ∗ in Figure 4 A). Second, a grouping strategy utilizing GPD grouping and a clustering based on phenotypes and chemicals with more relaxed thresholds of 0.75 (marked with $ in Figure 4 A) . Individual points represent groups exceeding the 95th percentile within their respective distribution (right-tail outliers).

S2.1 Distribution of CGPD tetramers and Chemicals across tissue groups

The distribution of CGPD tetramers and their underlying unique compounds provides insights into the tissue-specific and organism-specific representation of pesticides, pharmaceuticals, and bisphenols.

For **pesticides**, the nervous system consistently had the highest number of tetramers across all organisms, with rats having the largest count (5027 tetramers) compared to humans (3356) and mice (2824). This pattern was reflected in the number of unique pesticide compounds, with 44 in rats, 32 in humans, and 24 in mice. Additional tissues with substantial pesticide representation included the reproductive system in rats (1914 tetramers, 34 compounds) and the liver across all species.

Pharmaceuticals were extensively represented, with tetramers most abundant in the liver for humans (24179) and mice (23513) and in the nervous system for rats (41931). Unique compounds showed similar patterns, with the nervous system and liver consistently exhibiting high diversity across all organisms. Rats showed the highest chemical diversity in the nervous system (278 compounds), while humans and mice had comparable diversity in the liver and immune system.

Bisphenols showed fewer tetramers compared to pesticides and pharmaceuticals, with the reproductive system being the most frequently represented tissue group across all organisms (e.g., 10733 tetramers in rats). Despite the high number of tetramers, the number of unique bisphenol compounds was limited, ranging from 2 to 9 across tissues and organisms. Key tissues included the liver, fat tissues, and cardiovascular system, though their contributions were modest compared to other chemical classes.

These results emphasize significant differences in CGPD tetramer counts and unique compound diversity across chemical classes, tissues, and organisms. While pharmaceuticals demonstrated the highest coverage and diversity, pesticides and bisphenols were more limited in scope, particularly for unique compounds.

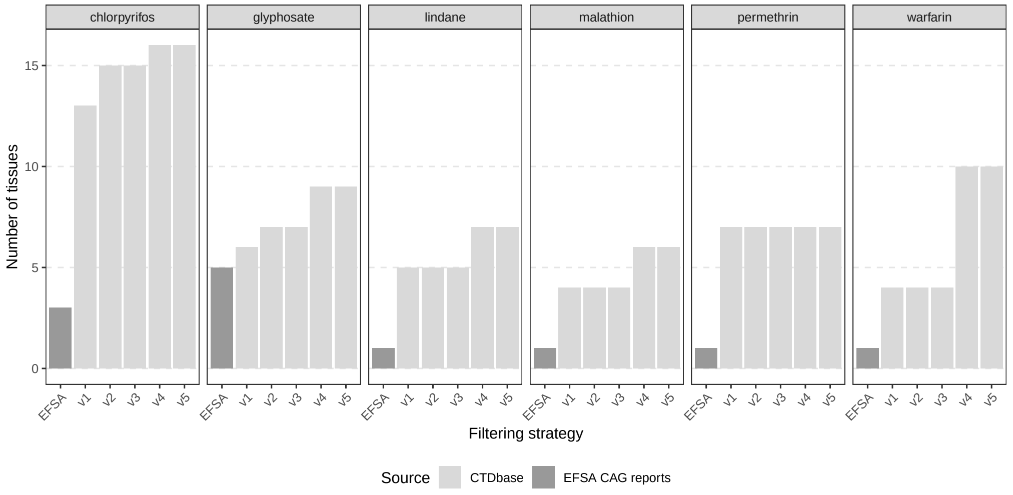

Fig. S7: Comparison of the number of affected tissues for six compounds with high discrepancy between EFSA CAG reports and `CTDbase`. The number of tissues is shown based on the original EFSA CAG report from 2012 and the five different filtering strategies to calculate CGPD tetramers (v1 to v5).

S3 Tissue-specific comparisons of EFSA CAG pesticides and `CTD` data

The six pesticides with the most significant discrepancies in tissue associations between the EFSA CAG reports and `CTDbase` include 2,4-D, metiram, thiamethoxam, ziram, chlorpyrifos, glyphosate, and warfarin. These pesticides highlight key differences in the number of tissues identified using CGPD tetramers compared to the EFSA CAG definitions.

Pesticides such as 2,4-D, metiram, thiamethoxam, and ziram were linked to a substantial number of tissues in the EFSA CAG reports (10, 7, 8, and 8, respectively) but only one or two tissues using `CTD` data. Conversely, for pesticides such as chlorpyrifos, glyphosate, and warfarin, we identified far more impacted tissues using CGPD tetramers than reported in the EFSA CAGs. Chlorpyrifos, for instance, was associated with 16 tissues in `CTDbase`, compared to just three in the EFSA CAG reports (eye, nervous system, and reproductive system). Glyphosate and warfarin were linked to 9 and 10 tissues, respectively, using CGPD tetramers, compared to 5 and 1 tissue in the EFSA reports.

Supplementary Figure S7 illustrates how different filtering strategies (v1–v5) influenced the identification of tissues for these six pesticides. Notably, warfarin exhibited the most dramatic increase in the number of impacted tissues under filtering strategy v5 compared to v1. This increase was primarily due to the omission of tissue specificity for disease interactions in v5, leading to associations with diseases like coronary artery disease and blood coagulation disorders. These diseases were linked to various tissues, including lung, muscles, spleen, and reproductive organs, based on `CTD`-annotated interactions.

Chlorpyrifos also showed an increase in the number of affected tissues from 13 in v1 to 16 in v5. In contrast, the EFSA CAG reports linked chlorpyrifos to only three tissues. The EFSA CAG classifications relied on data from pesticide databases, Draft Assessment Reports (DARs), and literature published before 2010, identifying approximately 2200 papers for chlorpyrifos. Of these, 169, 33, and 31 papers were linked to the liver, immune system, and kidney, respectively. It remains unclear whether these papers influenced the EFSA CAG focus on three target tissues or were disregarded due to insufficient evidence.

For liver associations, our CGPD tetramer approach linked chlorpyrifos to liver-specific diseases such as liver cirrhosis, fatty liver, hepatomegaly, and chemical-induced liver injury. These associations were supported by manually curated disease interactions with direct evidence, primarily from publications after 2017.

Table S6: Pesticides that were linked to the nervous system using CTD-derived data. Those compounds are mentioned in the CAG reports but not found to be toxic to the nervous system.

Chemical	MeSH ID	Chemical	MeSH ID
azoxystrobin	C087670	carbendazim	C006698
Chlordan	D002706	cyprodinil	C108338
Diquat	D004178	fenpyroximate	C415144
glyphosate	C010974	mepanipyrim	C104083
pyrachlostrobin	C513428	pyrimethanil	C108337
Warfarin	D014859		

Table S7: Pesticides that were linked to the liver using CTD-derived data. Those compounds are mentioned in the CAG reports but not found to be toxic to the liver.

Chemical	MeSH ID	Chemical	MeSH ID
1,2,4-triazole	C045575	bifenthrin	C099952
Chlorpyrifos	D004390	cyproconazole	C093628
Diazinon	D003976	Fenitrothion	D005278
Fenthion	D005284	fenvalerate	C017690
Hexachlorocyclohexane	D001556	Monocrotophos	D008999
sedaxane	C583365	Parathion	D010278
Permethrin	D026023	vinclozolin	C025643
Warfarin	D014859		

Table S8: Pesticides that were linked to the reproductive system using CTD-derived data. Those compounds are mentioned in the CAG reports but not found to be toxic to the reproductive system.

Chemical	MeSH ID	Chemical	MeSH ID
bifenthrin	C099952	Diquat	D004178
Endosulfan	D004726	Fenitrothion	D005278
Hexachlorocyclohexane	D001556	Malathion	D008294
Permethrin	D026023	vinclozolin	C025643

Table S9: Pesticides that were linked to the haematological system using CTD-derived data. Those compounds are mentioned in the CAG reports but not found to be toxic to the haematological system.

Chemical	MeSH ID	Chemical	MeSH ID
acephate	C001969	bifenthrin	C099952
carbendazim	C006698	Chlorpyrifos	D004390
cyhalothrin	C037304	cypermethrin	C017160
decamethrin	C017180	Diazinon	D003976
Dimethoate	D004117	Diquat	D004178
Endosulfan	D004726	enilconazole	C017435
ethion	C100038	Fenitrothion	D005278
fenvalerate	C017690	glyphosate	C010974
imidacloprid	C082359	Malathion	D008294
Permethrin	D026023	sulfoxaflor	C560328
Thiamethoxam	D000077922	Warfarin	D014859

Similarly, unspecific diseases such as fibrosis, necrosis, and edema were linked to the kidney based on recent publications, forming CGPD tetramers for chlorpyrifos in this organ. Direct evidence associating chlorpyrifos with "kidney diseases" was supported by four papers published from 2017 onwards.

These findings underscore the potential of CGPD tetramers to provide updated and more comprehensive tissue-specific pesticide associations based on recent curated evidence. They highlight discrepancies in historical datasets and the value of integrating newer data for refining cumulative risk assessments.

S4 Cluster characterization

Table S10: Phenotypes associated with the chemical cluster linked with Oligospermia. Here, we listed the number of unique chemical-phenotype interactions that are present in this cluster.

Phenotype name	Phenotype ID	# Chemical-Phenotype Interactions
spermatogenesis	GO:0007283	30
male gonad development	GO:0008584	6
ovarian follicle development	GO:0001541	6
antral ovarian follicle growth	GO:0001547	4
positive regulation of apoptotic process	GO:0043065	4
regulation of DNA-templated transcription	GO:0006355	3
Leydig cell differentiation	GO:0033327	2
positive regulation of epithelial cell proliferation	GO:0050679	2
regulation of transcription by RNA polymerase II	GO:0006357	2
Sertoli cell proliferation	GO:0060011	1
cell population proliferation	GO:0008283	1
decidualization	GO:0046697	1
negative regulation of cell population proliferation	GO:0008285	1
negative regulation of hydrolase activity	GO:0051346	1
nitric oxide biosynthetic process	GO:0006809	1
positive regulation of autophagy	GO:0010508	1
positive regulation of nitric oxide biosynthetic process	GO:0045429	1
response to estradiol	GO:0032355	1
uterus development	GO:0060065	1

Table S11: GO enrichment for eight genes associated with the cluster that was linked to the disease hyperglycemia in the haematological system in rats. The clusters were associated with the phenotypes glucose homeostasis (GO:0042593) and the positive regulation of insulin secretion (GO:0032024) and formed in filtering strategies v2 to v5. The eight genes were Gck (glucokinase), Il6 (interleukin 6), Ins1 (Insulin 1), Ins2 (insulin 2), Lepr (leptin receptor), Insr (insulin receptor), Lep (leptin), and Adipoq (adiponectin). We selected only those GO terms with a GeneRatio of 1, meaning that all 8 genes had to be present in the particular GO term.

GO term	Description	Count	Genes	GeneRatio	GO size	Go ratio
GO:0006006	glucose metabolic process	8	8	1	104	0.0769
GO:0019318	hexose metabolic process	8	8	1	107	0.0748
GO:0005996	monosaccharide metabolic process	8	8	1	113	0.0708
GO:0033500	carbohydrate homeostasis	8	8	1	170	0.0471
GO:0042593	glucose homeostasis	8	8	1	170	0.0471
GO:0005975	carbohydrate metabolic process	8	8	1	181	0.0442
GO:0032870	cellular response to hormone stimulus	8	8	1	356	0.0225
GO:0051050	positive regulation of transport	8	8	1	407	0.0197
GO:0048878	chemical homeostasis	8	8	1	427	0.0187
GO:0044281	small molecule metabolic process	8	8	1	474	0.0169

Table S12: Chemicals that were grouped and clustered in the rat haematological system. The group was linked with the disease hyperglycemia (MeSH: D006943) and the phenotypes glucose homeostasis (GO:0042593) and positive regulation of insulin secretion (GO:0032024). The 19 chemicals in the top were grouped using the filtering strategies v1 to v5. the other 12 chemicals at the bottom were grouped in this cluster in filtering strategies v2 to v5. A detailed list of the parameter settings is shown in Supplementary Table S13. The column 'Effect' denotes the effect of the compound w.r.t. blood glucose levels. A positive effects leads to decreasing and a negative effect leads to increasing glucose levels.

Filter	Chemical	MeSH ID	Group	Effect
v1 - v5	2,2',4,4'-tetrabromodiphenyl ether	C511295	Flame retardant	negative
	Acrylamide	D020106	Organic chemical	negative
	Arsenic	D001151	Heavy metal	negative
	Arsenic Trioxide	D000077237	Pharmaceutical	negative
	Atorvastatin	D000069059	Pharmaceutical	negative
	bisphenol A	C006780	Bisphenol, Pharmaceutical	negative
	Blood Glucose	D001786	Carbohydrate	negative
	Chlorpyrifos	D004390	Pesticide	negative
	dapagliflozin	C529054	Pharmaceutical	positive
	decabromobiphenyl ether	C010902	Flame retardant	negative
	Dexamethasone	D003907	Pharmaceutical	negative
	Dietary Fats	D004041	Lipid	negative
	Fructose	D005632	Carbohydrate, Pharmaceutical	negative
	Glucose	D005947	Carbohydrate, Pharmaceutical	negative
	imidacloprid	C082359	Pesticide	negative
	Letrozole	D000077289	Pharmaceutical	negative
	Nicotine	D009538	Pesticide	negative
	sodium arsenite	C017947	Pharmaceutical	negative
	Streptozocin	D013311	Pharmaceutical	negative
v2 - v5	acephate	C001969	Pesticide	negative
	cobaltiprotoporphyrin	C007095	Heterocyclic compounds	positive
	corilagin	C049096	Organic chemical	positive
	Curcumin	D003474	Pharmaceutical	positive
	Diazinon	D003976	Pesticide	negative
	Fatty Acids, Omega-3	D015525	Lipid, Pharmaceutical	negative
	Glyburide	D005905	Pharmaceutical	positive
	Lead	D007854	Heavy metal	negative
	Metformin	D008687	Pharmaceutical	positive
	Propolis	D011429	Mixture, Antimicrobial	positive
	Quercetin	D011794	Pharmaceutical	positive
	Resveratrol	D000077185	Pharmaceutical	positive

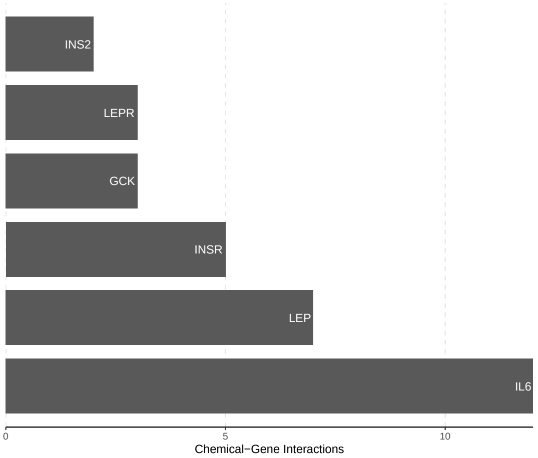

(a) Filtering strategy v1

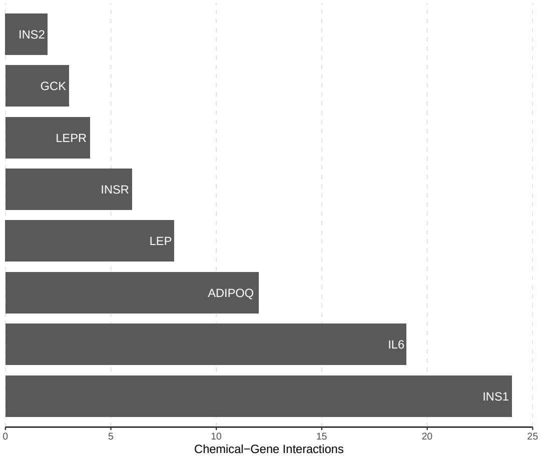

(b) Filtering strategies v2 - v5

Fig. S8: **Chemical-gene interactions in Hyperglycemia associated cluster.** (a) Unique chemical-gene interactions for the six associated genes in the chemical cluster formed in filtering strategy v1 containing 19 chemicals. (a) Unique chemical-gene interactions for the eight associated genes in the chemical cluster formed in filtering strategies v2-v5 containing 31 chemicals.

Table S13: Clusters containing chemicals that were linked to the disease hyperglycemia (MeSH: D006943) in the Haematological system of *Rattus norvegicus*. In total, 40 different parameter settings in filtering strategies v2 to v5 resulted in clusters with the same set of 31 chemicals. 10 different parameter settings in filtering strategy v1 resulted in a reduced cluster with 19 chemicals. The chemicals are listed in Supplementary Table S12

Filter	Grouping	Phenotype clustering		Chemical clustering		Chemicals
		Applied	Cutoff	Applied	Cutoff	
v1	pd	-	-	x	0.75	19
v1	pd	-	-	x	0.9	19
v1	pd	x	0.75	x	0.5	19
v1	pd	x	0.75	x	0.75	19
v1	pd	x	0.75	x	0.9	19
v1	pd	x	0.9	x	0.5	19
v1	pd	x	0.9	x	0.75	19
v1	pd	x	0.9	x	0.9	19
v1	pd	x	0.75	-	-	19
v1	pd	x	0.9	-	-	19
v2	pd	-	-	x	0.75	31
v2	pd	-	-	x	0.9	31
v2	pd	x	0.75	x	0.5	31
v2	pd	x	0.75	x	0.75	31
v2	pd	x	0.75	x	0.9	31
v2	pd	x	0.9	x	0.5	31
v2	pd	x	0.9	x	0.75	31
v2	pd	x	0.9	x	0.9	31
v2	pd	x	0.75	-	-	31
v2	pd	x	0.9	-	-	31
v3	pd	-	-	x	0.75	31
v3	pd	-	-	x	0.9	31
v3	pd	x	0.75	x	0.5	31
v3	pd	x	0.75	x	0.75	31
v3	pd	x	0.75	x	0.9	31
v3	pd	x	0.9	x	0.5	31
v3	pd	x	0.9	x	0.75	31
v3	pd	x	0.9	x	0.9	31
v3	pd	x	0.75	-	-	31
v3	pd	x	0.9	-	-	31
v4	pd	-	-	x	0.75	31
v4	pd	-	-	x	0.9	31
v4	pd	x	0.75	x	0.5	31
v4	pd	x	0.75	x	0.75	31
v4	pd	x	0.75	x	0.9	31
v4	pd	x	0.9	x	0.5	31
v4	pd	x	0.9	x	0.75	31
v4	pd	x	0.9	x	0.9	31
v4	pd	x	0.75	-	-	31
v4	pd	x	0.9	-	-	31
v5	pd	-	-	x	0.75	31
v5	pd	-	-	x	0.9	31
v5	pd	x	0.75	x	0.5	31
v5	pd	x	0.75	x	0.75	31
v5	pd	x	0.75	x	0.9	31
v5	pd	x	0.9	x	0.5	31
v5	pd	x	0.9	x	0.75	31
v5	pd	x	0.9	x	0.9	31
v5	pd	x	0.75	-	-	31
v5	pd	x	0.9	-	-	31

References

Nielsen E, Norhede P, Boberg J, Krag Isling L, Kroghsbo S, Hadrup N, Bredsdorff L, Mortensen A, Larsen JC (2012) Identification of Cumulative Assessment Groups of Pesticides. EFSA Supporting Publications 9, DOI https://doi.org/10.2903/sp.efsa.2012.EN-269
